# Review of Linear-Array-Transducer-Based Volumetric Ultrasound Imaging Techniques and Their Biomedical Applications

**DOI:** 10.3390/bioengineering12090906

**Published:** 2025-08-23

**Authors:** Ninjbadgar Tsedendamba, Yuon Song, Eun-Yeong Park, Jeesu Kim

**Affiliations:** 1 Departments of Cogno-Mechatronics Engineering, Pusan National University, Busan 46241, Republic of Korea; ninjee97@pusan.ac.kr (N.T.); yuonsong@pusan.ac.kr (Y.S.); 2 Department of Bio and Brain Engineering, Korea Advanced Institute of Science and Technology, Daejeon 34141, Republic of Korea

**Keywords:** ultrasound, linear-array, volumetric imaging, biomedical imaging

## Abstract

Ultrasound imaging is one of the most widespread biomedical imaging techniques thanks to its advantages such as being non-invasive, portable, non-ionizing, and cost-effective. Ultrasound imaging generally provides two-dimensional cross-sectional images, but the quality and interpretative ability vary based on the experience of the examiner, leading to a lack of objectivity and accuracy. To address these issues, there is a growing demand for three-dimensional ultrasound imaging. Among the various types of transducers used to obtain three-dimensional ultrasound images, this paper focuses on the most standardized probe, the linear array transducer, and provides an overview of the system implementations, imaging results, and applications of volumetric ultrasound imaging from the perspective of scanning methods. Through this comprehensive review, future researchers will gain insights into the advantages and disadvantages of various approaches to three-dimensional imaging systems using linear arrays, providing direction and applicability for system configuration and application.

## 1. Introduction

Ultrasound imaging (USI) is a widely utilized biomedical imaging technique that enables the non-invasive and non-ionizing visualization of internal anatomical structures by achieving pulse-echo signals of ultrasound (US) waves propagating through biological tissues [1,2]. Compared to other conventional clinical imaging techniques such as magnetic resonance imaging (MRI), positron emission tomography (PET), and X-ray computed tomography (CT), USI offers distinct advantages of temporal resolution and accessibility for routine monitoring [3,4,5]. By visualizing the structural information of various organs, USI is extensively employed across a broad range of clinical applications, ranging from diagnostic assessments to image-guided interventions [6,7,8,9].

Conventional USI primarily produces two-dimensional (2D) B-mode images by using various types of one-dimensional (1D) array transducers [10,11,12]. These cross-sectional images offer real-time morphology of biological tissues with hand-held operation of the examiner. While highly effective in many clinical scenarios, 2D imaging constrains diagnostic interpretation and reproducibility, especially when anatomical structures extend beyond the imaging plane or exhibit a complex three-dimensional (3D) morphology. These limitations have motivated the development of 3D volumetric USI [13,14,15], which provides more comprehensive and intuitive anatomical information by capturing entire volumes rather than single slices [16,17,18].

For 3D USI, various geometries of US transducers have been utilized [19,20]. The most straightforward approach involves mechanically scanning a single-element transducer, which remains attractive due to its simplicity and low cost. However, it suffers from slow acquisition and motion artifacts, which thus restrict its clinical applicability [21,22,23]. Alternately, 2D matrix array transducers enable real-time volumetric imaging through full electronic steering in both azimuthal and elevational dimensions [24,25,26], and have found applications in fields such as obstetric imaging [27,28,29]. However, their widespread adoption is limited by technological complexity, reduced frame rates, and high fabrication costs. Ultrasound computed tomography (USCT) represents another paradigm, reconstructing 3D images using inverse algorithms from multi-angle transmission data [30]. USCT offers full-angle volumetric reconstructions and quantitative imaging potential, particularly for breast imaging, but typically requires extensive hardware infrastructure and prolonged acquisition, limiting its real-time applicability.

**Figure 1:**
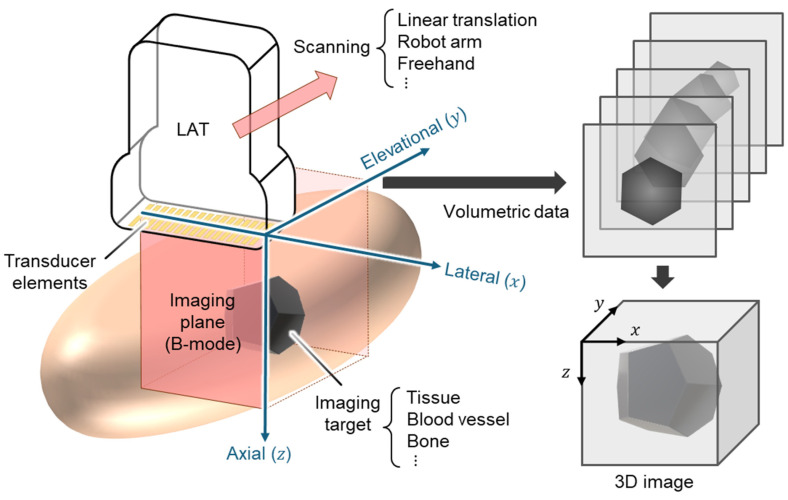
Schematic illustration for 3D ultrasound image generation using LAT. LAT, linear array transducer; 3D, three-dimensional.

Among these approaches, linear-array-based volumetric USI has emerged as a highly practical and adaptable solution. Linear array transducers (LATs) are already ubiquitous in clinical settings and benefit from mature fabrication processes, standardized interfaces, and broad system compatibility. LATs are composed of numerous small piezoelectric elements (typically 128–256) arranged linearly (Figure 1). The acoustic beamlines generated by LATs produce rectangular-shaped B-mode images with uniform beam density. This configuration offers an excellent near-field resolution, particularly advantageous for imaging superficial anatomical structures. LAT-based USI conventionally generates cross-sectional (x×z) B-mode images. To reconstruct 3D volumes, additional scanning in the elevational (y) direction is required. By integrating advanced scanning strategies, LATs can be repurposed for 3D image acquisition with relatively modest hardware modifications.

Compared to single-element approaches, LATs offer much faster acquisition and superior image quality thanks to simultaneous multi-line reception and dynamic focusing. Compared to 2D matrix arrays, LAT-based systems require an order of magnitude fewer channels, allowing for simpler electronics and higher native frame rates. While matrix arrays can capture a full volume instantaneously without mechanical motion, they are constrained by reduced temporal resolution from channel multiplexing and higher manufacturing complexity. In contrast, LAT-based volumetric imaging offers a cost-effective and clinically accessible solution, achieving comparable in-plane resolution and adequate volumetric coverage for many applications. These attributes make LAT-based volumetric USI a compelling platform for both translational research and widespread clinical adoption.

In general, the imaging depth and spatial resolution in USI are highly dependent on the acoustic frequency. Acoustic attenuation in biological tissue increases approximately linearly with frequency and can be expressed as follows [31,32]:

(1) $$\alpha \left(f\right)\approx \alpha_{0}f^{n},$$

where $\alpha \left(f\right)$ is the acoustic attenuation coefficient at frequency f. $\alpha_{0}$ and n≈1 are attenuation constants related to the tissue. The intensity of a US wave $I\left(z\right)$ decreases exponentially from the initial intensity $I_{0}$, according to propagation distance z as follows:

(2) $$I\left(z\right)=I_{0}e^{-a\left(f\right)\cdot z}.$$

Higher frequencies reduce the effective imaging depth due to increased attenuation, but their shorter wavelength enables finer axial and lateral resolution. Conversely, lower frequencies allow for deeper penetration by sacrificing imaging resolution. Typical LATs operate in the frequency range of 1–20 MHz, which provides a suitable balance for clinical human imaging, with a penetration depth of several centimeters and spatial resolutions on the order of a few hundred micrometers.

Moreover, LAT platforms exhibit exceptional expandability toward functional and molecular imaging modalities. They can be readily adapted to support photoacoustic imaging (PAI), which leverages light absorption-induced acoustic signals to provide functional and molecular information [33,34,35,36,37]. Based on the similarity in signal detection the and image generation processes of PAI and USI, the two modalities are often configured in a single imaging platform [38,39]. Particularly, when integrated with LAT-based systems, PAI enables simultaneous structural and molecular imaging, offering valuable insights into tissue physiology, angiogenesis, and tumor microenvironments [40,41], showing potential for visualizing the functional information of biological tissues based on the multispectral molecular absorption characteristics [42,43].

Likewise, ultrafast US Doppler (UFD) imaging, which can visualize blood flow dynamics with an exceptionally high temporal resolution, is also readily implemented on LAT platforms by leveraging high-frame-rate plane wave imaging [44,45,46]. Unlike conventional Doppler imaging, which relies on sequential line-by-line scanning and suffers from limited frame rates, UFD achieves frame rates in the order of thousands of frames per second by utilizing plane wave transmission with diverged angles, enabling fast image acquisition across the entire imaging field of view [47]. This enables the detection of slow-flow vessels, microcirculations, and transient hemodynamic changes with high temporal and spatial resolution. When applied in conjunction with LAT-based volumetric acquisition, UFD allows for 3D functional imaging of perfusion, vascular reactivity, and cardiac mechanics.

This review presents a comprehensive overview of volumetric USI techniques based on LATs. The system configurations, volumetric scanning strategies, representative imaging results, and biomedical applications are explored. This review specifically investigates three representative scanning mechanisms for achieving volumetric data acquisition with LATs: linear translational scanning, robot-arm-assisted scanning, and freehand scanning. By summarizing the imaging results, the strengths and limitations of each approach can be explored. This article aims to guide researchers and clinicians toward a deeper understanding of the current state and future directions of LAT-based 3D USI, highlighting its potential for both clinical and preclinical imaging applications.

## 2. Linear Translational Scanning

**Figure 2:**
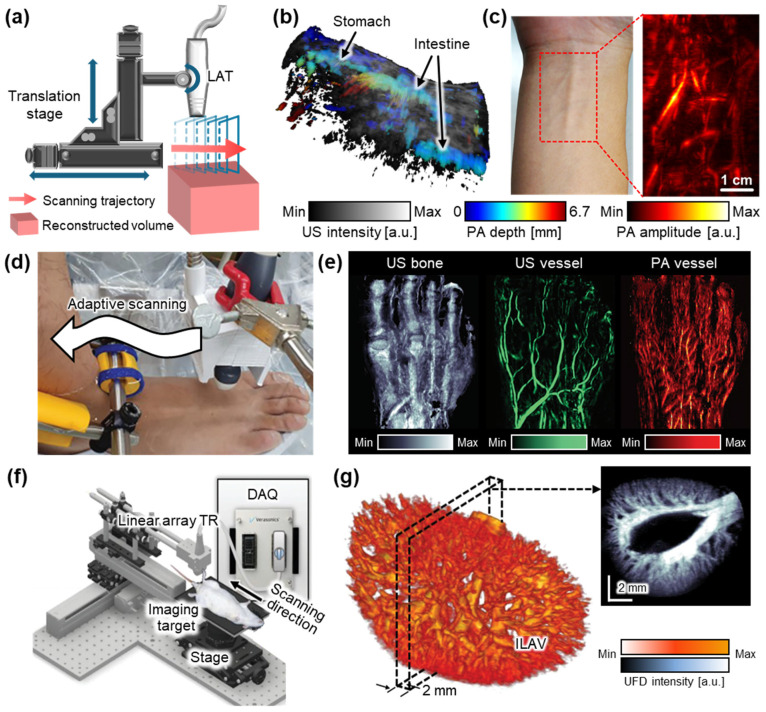
Representative 3D US imaging results using the linear translational scanning mechanism. (**a**) Schematic illustration of the motorized translational scanning for 3D US imaging. (**b**) Volumetric PA and US overlaid image of the rat abdomen area. Color represents the depth of the PA signals. (**c**) PA image of human forearm. (**d**) Photograph of the adaptive scanning module to achieve 3D data of peripheral vasculatures. (**e**) Structure and blood vessel network acquired from the human foot. (**f**) Schematic illustration of a 3D UFD imaging system using a linear translation stage. (**g**) 3D structure of a rat kidney renal vascular network, reconstructed from 3D UFD. 3D, three-dimensional; US, ultrasound; PA, photoacoustic; UFD, ultrafast ultrasound Doppler; TR, transducer; FB, fiber bundle; GP, gel pad; DAQ, data acquisition model; and ILAV, interlobular artery–vein. The images are adapted with permission from [50,52,56].

Linear translational scanning is one of the most fundamental and widely adopted methods for acquiring volumetric data using LATs [48,49]. In this approach, the LAT is mechanically translated along the elevational (y) axis, orthogonal to the imaging plane (x×z), to sequentially acquire a series of 2D slices across a predefined cuboidal volume (Figure 2a). Because the scanning trajectory and speed are predefined and controllable, the spatial position of each cross-sectional image can be accurately determined, making the image generation procedure straightforward. The key advantages of linear translational scanning are simplicity and reproducibility. The motorized translation ensures consistent probe orientation throughout the acquisition, minimizing geometric distortion and operator-dependent variability. Furthermore, the method is relatively easy to implement, requiring minimal modification of existing 2D imaging systems. Therefore, this approach has been widely utilized as an initial strategy for volumetric data acquisition, especially for functional imaging modalities, including PAI and UFD (Table 1).

**Table 1:** Summary of three-dimensional US imaging systems with the linear translational scanning of LATs. US, ultrasound; PA, photoacoustic; UFD, ultrafast US Doppler; LAT, linear array transducer; $f_{C}$, center frequency of LAT; $f_{BW}$, bandwidth of LAT; and $n_{E}$, number of elements.

Scanning		Transducer			Imaging Mode	Application	Ref.
Volume [mm^3^]	Time [s]	$f_{C}$ **[MHz]**	$f_{BW}$ **[MHz]**	$n_{E}$			
40 × 75 × 30 *	30 *	8.5	3–12	128	US, PA	Rat gastrointestinal tract, human forearm	[50]
40 × 50 × 24 *	25 *	8.5	3–12	128	US, PA	Mouse gastrointestinal tract	[51]
114 × 160 × 30 *	211	8.5	3–12	128	US, PA	Human foot	[52]
38.4 × 40 × 30	20	8.5	3–12	128	US, PA	Human neck, wrist, thigh	[53]
38.1 × 31.4 × 25 *	11.4	8.5	3–12	128	US, PA	Human melanoma	[54]
12.8 × 12.8 × 20	1200	15	–	128	US, UFD	Mouse brain	[55]
24 × 12.8 × 15	840	18	14–22	128	US, UFD	Rat kidney	[56]
6 × 6 × 6	1.5	18	14–22	128	US, UFD	Mouse brain	[57]
100 × 100 × 120 *	75 *	7.5	3–10	192	US	Human residual limbs	[58]
128 × 180 × 180 *	–	3.5	2–4.75	128	US	Breast phantom	[59]
128 × 180 × 180	180	3.5	2–4.75	128	US	Breast phantom	[60]
100 × 100 × 250	9.4	5	4–7	128	US	Human hand, wrist, forearm	[61]

* Calculated from values provided in the literature. – not reported in the original publication.

Kim et al. demonstrated a dual-modal PA and US imaging platform by integrating a programmable US machine with a transportable laser source [50]. The system employed a 128-element LAT with a center frequency of 7.5 MHz and a laser operating at a pulse repetition frequency of 10 Hz. During translational scanning, both PA and US data were acquired. Overlaid PA/US images from rat abdomens (Figure 2b) and the human forearm (Figure 2c) successfully visualized both molecular absorption contrast and structural features, demonstrating the potential of the system for clinical and preclinical applications. In a following study, the same platform was further applied to investigate the biodistribution of exogenous contrast agents in small animals, achieving deep tissue imaging at depths of up to 4.6 cm, with theoretical axial resolutions of 0.23 mm and 0.14 mm for PA and US, respectively [51]. The resulting images clearly delineated contrast-enhanced signals from internal organs such as the bladder and gastrointestinal tract.

Choi et al. proposed a translational scanning method that follows the contours of the skin surface using a two-phase adaptive scanning strategy (Figure 2d) [52]. Volumetric PA/US data were acquired from the feet of six healthy volunteers, with each foot scanned in two stages. The first phase rapidly identified the skin surface, while the second phase adaptively adjusted the axial position of the probe for maintaining a consistent probe-to-skin distance during scanning. The total acquisition time per volumetric dataset was approximately 197 s, demonstrating feasibility for clinical use. The repeatability of this method was validated through five repeated scans, which consistently visualized internal structures and multilayered vascular networks. Furthermore, the system enabled the quantitative assessment of vascular morphology and hemoglobin oxygenation, underscoring its potential for evaluating peripheral vascular diseases (Figure 2e).

Despite these advancements, the bulkiness and mechanical complexity of traditional linear translation stages remain a barrier to broader clinical adoption. To address this limitation, Lee et al. developed a hand-held linear translation scanner to achieve volumetric data [53]. This compact imaging device, measuring 100 × 80 × 100 mm^3^ and weighing 950 g, integrated a 128-element LAT and a miniaturized motorized scanner based on a Scotch yoke mechanism. By achieving an approximately 0.2 mm axial resolution at a depth of 30 mm, the platform was successfully applied to the in vivo imaging of blood vessels in the neck, wrist, and thigh. Its clinical utility was further demonstrated through the non-invasive visualization of cutaneous melanoma lesions in human subjects in vivo [54]. The system achieved depth-resolved PA and US images, enabling the measurement of the depth of lesions with a mean absolute error of 0.36 mm. The measured depth was validated by comparing it to histopathological measurements, supporting its accuracy for tumor thickness assessment.

Linear translational scanning has also been applied to UFD imaging for 3D visualization of the vasculature network. Demené et al. employed a multi-axis scanning system composed of a 3-axis linear stage and a 1-axis yaw stage to acquire volumetric data [55]. By using a 15 MHz LAT that transmitted eight compounded plane waves at a frame rate of 800 Hz, their system was able to achieve UFD images at depths of up to 20 mm. A spatiotemporal singular value decomposition (SVD) filter was applied to suppress tissue signals and enhance microvascular contrast. The approach was validated through whole-brain imaging in rats, successfully revealing cerebral microvasculature and blood flow dynamics relevant to neurovascular studies. However, the relatively long (~20 min for 12.8 mm range) scanning time limits its potential feasibility in biomedical applications. To improve the data acquisition speed, Oh et al. introduced a high-frame-rate system operating at 1 kHz, utilizing seven compounded plane waves to generate volumetric vascular images (Figure 2f) [56]. Volumetric imaging was performed using an LAT with a center frequency of 18 MHz. They implemented accelerated image processing, enabling UFD data acquisition and image generation in 7 s for a single B-mode frame. This reduced the total acquisition time to ~14 min for a 24 mm elevational range with a step size of 0.2 mm (Figure 2g). Despite this improvement, the system remained insufficient for real-time imaging. To further accelerate 3D imaging, Generowicz et al. demonstrated a sweep motion scanning approach for UFD imaging of the mouse brain [57]. Their system transmitted eight compounded plane waves at a frame rate of 4 kHz while performing a continuous back-and-forth sweep across a 6 mm region in the elevational direction. This setup enabled volumetric imaging at a rate of one volume every 1.5 s, significantly enhancing temporal resolution compared to conventional stepwise scanning methods.

**Figure 3:**
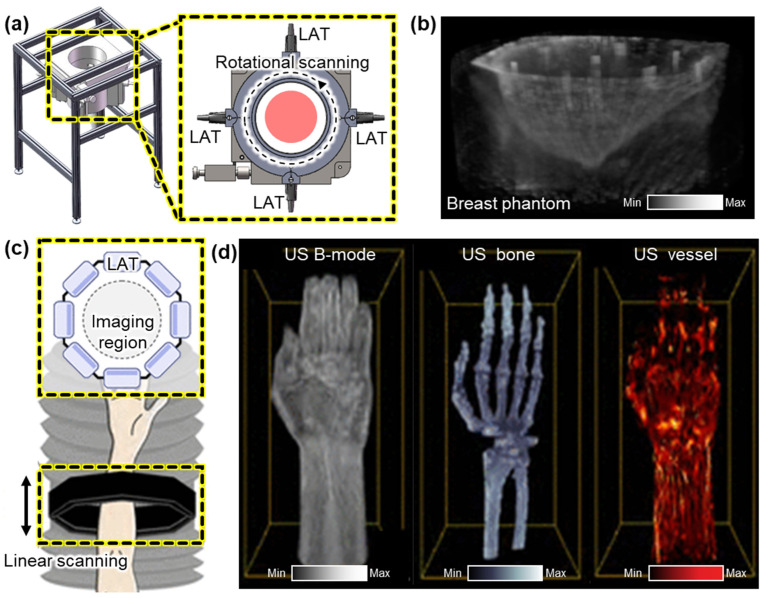
Representative 3D US imaging results using multiple LATs. (**a**) Schematic illustration of a 3D US imaging system using rotational scanning of four LATs. (**b**) The resulting 3D volume of a phantom that mimics a human breast. (**c**) Schematic of a 3D US imaging system using linear scanning of eight LATs. (**d**) 3D imaging results of the human forearm area, demonstrating volumetric visualization of skin surface, bone, and blood vessels. 3D, three-dimensional; US, ultrasound; and LAT, linear array transducer. The images are adapted with permission from [59,60,61].

While linear translational scanning has demonstrated promising results for 3D volume imaging, it is inherently limited in tomographic image generation due to its unidirectional acquisition geometry. To overcome this constraint, rotational scanning approaches have been introduced to achieve more complete angular sampling and improve spatial resolution. Ranger et al. demonstrated a volumetric US tomography system that utilizes the rotational scanning of a LAT [58]. To achieve full volumetric coverage, the system applied additional linear vertical translation, enabling the acquisition of a stack of 2D slices over a cylindrical imaging volume. A camera-based motion tracking system was integrated to monitor the position and orientation of the probe in real time, allowing for the motion-compensated alignment of the acquired slices. They successfully acquired volumetric US images from the residual limb of human subjects. The accuracy of the resulting 3D ultrasound volumes was validated by showing a good correlation with corresponding MRI scans. Liu et al. also demonstrated the rotational scanning of LATs [59]. Their system is configured with four 128-element LATs arranged at 90° intervals around the target (Figure 3a). This setup reduced the required scanning rotation range to 90°, significantly minimizing the acquisition time. Each pair of opposing transducers enabled the simultaneous acquisition of reflected and transmitted ultrasound signals. This geometry enhanced spatial resolution and contrast by capturing multi-angle perspectives. In a subsequent study, the same group demonstrated the multi-perspective imaging of breast phantoms containing multiple masses of varying sizes [60]. By reconstructing both vertical and horizontal cross-sections, the system successfully visualized the volumetric structure of the phantoms (Figure 3b).

To further improve imaging speed and eliminate the need for mechanical rotation, Park et al. proposed an octagonal array configuration composed of eight LATs arranged to form a 1024-element circular aperture with a diameter of 162 mm [61]. This geometry enabled full-angle signal acquisition without rotating the probe, providing an isotropic volumetric spatial resolution of less than 0.05 mm. The entire assembly was mounted on a motorized scanner and vertically translated into a water tank to acquire volumetric data (Figure 3c). To address computational challenges, a partial beamforming strategy was implemented. The local beamforming of data from each LAT was performed prior to transmitting data to a central data processing unit. This significantly reduced data transfer and reconstruction latency, enabling real-time volumetric imaging at 25 Hz, which is suitable for in vivo applications. Using this system, they successfully imaged the human forearm, clearly visualizing skin layers, skeletal structures, and vascular networks (Figure 3d).

## 3. Robot Arm Scanning

**Figure 4:**
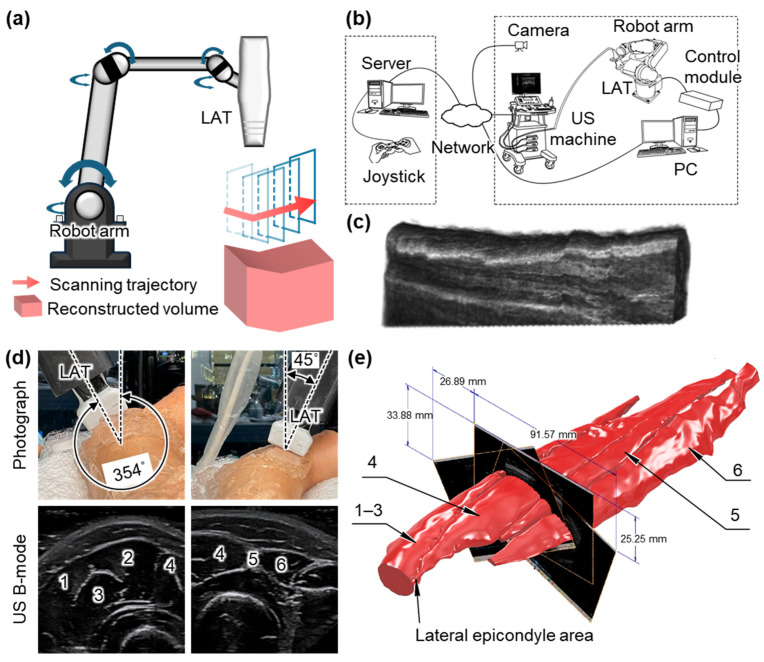
Representative 3D US imaging results using the robot arm mechanism. (**a**) Schematic illustration of the robot arm scanning for 3D US imaging. (**b**) Schematic illustration of a 3D US imaging system using a remotely controlled robot arm scanner. (**c**) The resulting 3D image of the human forearm. (**d**) Photograph and resulting US B-mode images of the human forearm achieved via a robot arm scanning system. (**e**) Reconstructed volumetric model of the forearm tissues in static contraction. 1, brachioradialis muscle. 2, extensor carpi radialis longus muscle. 3, extensor carpi radialis brevis muscle. 4, extensor digitorum muscle. 5, extensor digiti minimi muscle. 6, extensor carpi ulnaris muscle. 3D, three-dimensional; US, ultrasound; LAT, linear array transducer; and PC, personal computer. The images are adapted with permission from [62,66].

While linear translational scanning has been applied for 3D USI, applying this scanning mechanism in the human body may be limited because the skin surface is not flat. With advances in robotic technology, robot arms are increasingly being integrated into medical procedures and diagnostic imaging. In medical imaging, these systems offer consistent and precise control of probe motion, maintaining stable contact with the skin and following predefined trajectories (Figure 4a). By employing more than six degrees of freedom (DOFs), robot arms can provide reproducible and robust scanning while maintaining close contact on the surface of the curved samples (Table 2).

**Table 2:** Summary of three-dimensional US imaging systems with robot arm scanning of LATs. US, ultrasound; LAT, linear array transducer; $f_{C}$, center frequency of LAT; $f_{BW}$, bandwidth of LAT; and $n_{E}$, number of elements.

Scanning		Transducer			Imaging Mode	Application	Ref.
Volume [mm^3^]	Time [s]	$f_{C}$ **[MHz]**	$f_{BW}$ **[MHz]**	$n_{E}$			
–	–	7.5	4–9	128	US	Fetus phantom	[62]
10 × 100 × 20	–	10	5–12	192	US	Vascular phantom	[63]
–	–	10	5–14	128	US	Human thyroid lobe	[64]
–	–	–	4–12	192	US	Thyroid phantom	[65]
–	–	13	–	–	US	Forearm phantom	[66]
–	–	12	–	256	US	Human carotid artery	[67]
–	–	–	5–9	–	US	Human thyroid	[68]

– not reported in the original publication.

Huang et al. demonstrated a robot-assisted system for 3D USI using a 6-DOF robotic arm, which was manually controlled by a joystick with visual feedback from four external cameras (Figure 4b) [62]. The system was first validated on phantoms and then applied to obtain 3D US images of the human forearm (Figure 4c). The system exhibited high computational efficiency, reconstructing 290 frames with a scanning time of 162 s and a reconstruction time of ~8 s for generating a volume image. While the results confirmed the feasibility of robot-assisted 3D imaging, the reliance on manual operation limited consistency across repeated scans. To address this limitation, Janvier et al. proposed a robotic scanning method capable of learning and replaying predefined probe trajectories [63]. In their system, a 6-DOF robot arm equipped with a 10 MHz LAT performed controlled linear scans using a two-phase process: a manual “teach” phase that records the scanning trajectory during manual movement and a “replay” phase that reproduces the recorded scanning motion. This ensured consistent probe orientation and motion throughout the scan. The method was validated using a vascular-mimicking phantom with two stenotic lesions, and the reconstructed 3D volumes clearly visualized both regions of narrowing with high accuracy and repeatability. However, the reliance on fixed trajectories may restrict adaptability to patient-specific anatomies in clinical practice.

Kojcev et al. introduced an automated scanning mechanism based on surface detection [64]. Prior to scanning, a 3D camera captured the skin surface, and the user defined the imaging region. The system then automatically planned and executed a sweeping trajectory, maintaining optimal contact and orientation throughout the scan. During acquisition, both US images and tracking data were recorded and compounded into a volumetric dataset. Validation on four healthy volunteers showed reduced variability in thyroid lobe measurements compared to expert-operated scans, highlighting the improved repeatability. Similarly, Zielke et al. demonstrated a robotic scanning system for thyroid imaging that operated without reliance on external tracking systems [65]. Their path control algorithm allowed the robot to autonomously perform linear sweeps over the thyroid region. Using built-in force sensors, the system maintained consistent contact pressure and orientation, enabling the accurate acquisition of coronal, axial, and 3D views with anatomical details. Kapravchuk et al. also reported a sensor-integrated scanning platform that employed a 6-DOF robotic arm equipped with a force–torque sensor to regulate contact pressure during probe motion [66]. The LAT was guided along predefined trajectories (Figure 4d), minimizing tissue deformation artifacts and enhancing spatial accuracy. The system was tested on forearm-mimicking phantoms with known geometries. The resulting 3D reconstructions showed volume errors below 1% and a spatial resolution of 0.2 mm, confirming the accuracy and reliability of the method (Figure 4e).

More recently, Huang et al. proposed a fully autonomous carotid artery imaging system that eliminates the need for human intervention throughout the scanning process [67]. In the pre-scan phase, the robotic system plans and executes a scanning trajectory based on anatomical landmarks. A U-Net-based segmentation model provided real-time guidance for probe positioning and contact force adjustment. The system achieved an 86.1% success rate in capturing volumetric carotid artery images in human subjects. Zhou et al. proposed a fully autonomous thyroid imaging system using a 7-DOF robotic arm guided by multimodal imaging and deep learning [68]. They added flexibility, allowing for better adaptation to curved surfaces. The scanning process begins with RGB-D imaging to localize the neck region and guide the initial probe placement. A deep-learning-based controller then adapts the scanning trajectory using US feedback, actively avoiding acoustic shadows. This strategy ensures safe and stable contact, providing highly accurate operator-free volumetric thyroid imaging with a tracking accuracy of 98.6%.

## 4. Freehand Scanning

**Figure 5:**
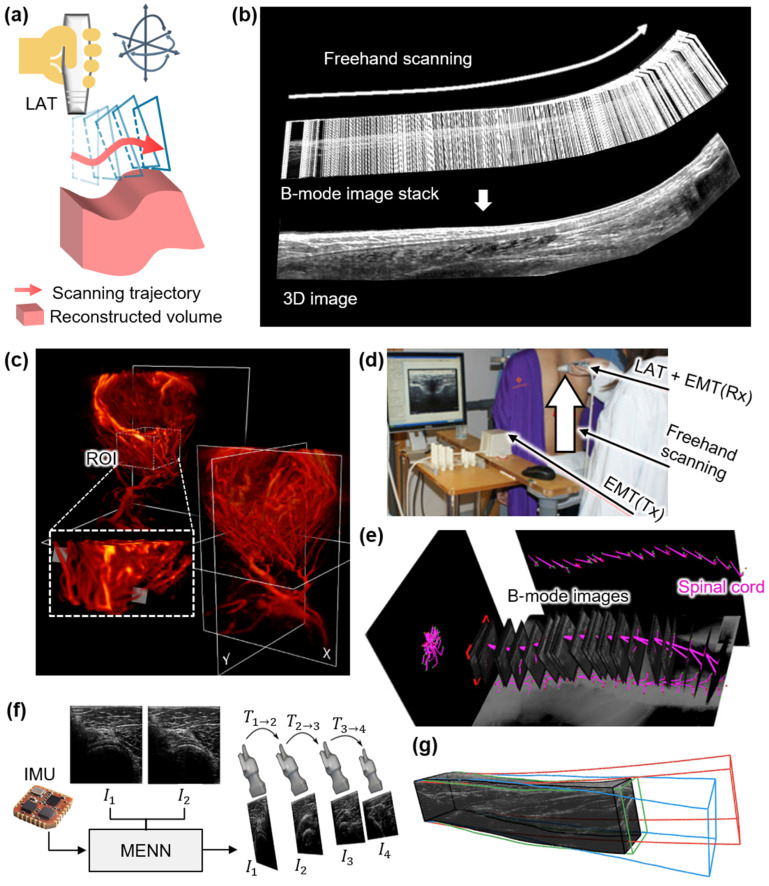
Representative 3D US imaging results using the freehand mechanism. (**a**) Schematic illustration of the freehand scanning for 3D US imaging. (**b**) Stacked B-mode images from freehand scanning and the representative cross-section image of the reconstructed 3D US volume of the human Achilles tendon. (**c**) 3D UFD imaging of a frontoparietal glioblastoma multiforme and the lateral ventricles. (**d**) Photograph of a freehand scanning tracked via an EMT module. (**e**) The achieved B-mode images. The purple lines are segmented spinal cords featured in the B-mode images. (**f**) Schematic diagram of frame-to-frame estimation utilizing the MENN. (**g**) The comparison between reconstruction methods: black, reference trajectory tracked by an optical tracking module; red, linear motion; blue, speckle decorrelation; and green, proposed MENN. 3D, three-dimensional; US, ultrasound; LAT, linear array transducer; EMT, electromagnetic tracer; Tx, transmitter; Rx, receiver; IMU, inertial measurement unit; MENN, motion estimation neural network; I, image; and T, transformation matrix between images. The images are adapted with permission from [70,72,73,77].

Freehand scanning stacks B-mode images by tracking the position and angles of the probe, allowing the user to freely set a scan path (Figure 5a). Freehand scanning provides maximum DOF during image acquisition by allowing operators to manually control the scanning direction and area. This flexibility enables the adaptive scanning of anatomical variations. However, the accurate reconstruction of 3D volumes from 2D B-mode images requires precise knowledge of the position and orientation of the imaging probe at each acquisition frame. In freehand scanning, the scanning motion is recorded via various types of tracking modules that are attached to the imaging probe (Table 3).

**Table 3:** Summary of three-dimensional US imaging systems with the freehand scanning of LATs. US, ultrasound; PA, photoacoustic; UFD, ultrafast US Doppler; LAT, linear array transducer; $f_{C}$, center frequency of LAT; $f_{BW}$, bandwidth of LAT; and $n_{E}$, number of elements.

Scanning		Transducer			Imaging Mode	Application	Ref.
Volume [mm^3^]	Time [s]	$f_{C}$ **[MHz]**	$f_{BW}$ **[MHz]**	$n_{E}$			
350 × 50 × 200	–	9.5 *	5–14 *	128 *	US	Fetus phantom	[69]
20 × 15 × 120	15–20	10	5–15 *	128 *	US	Human Achilles tendon	[70]
–	60 *	7.5 *	5–12 *	128 *	US	Carotid artery phantom	[71]
–	–	5.3 7.8	–	192 168	US, UFD	Human brain	[72]
–	120	10 *	6–14	128 *	US	Human spine	[73]
8 × 8 × 8 *	3	10	6–14 *	128	US Elastography	Elasticity phantom	[74]
–	90	8.5 *	5–12 *	192 *	US	Fetal phantom	[75]
100 × 70 × 60	–	14 *	13–15	–	US	Breast phantom	[76]
–	–	5	–	128	US	Human forearm	[77]
46 × 83 × 55	9	8 *	4–12 *	–	US	Human carotid artery	[78]
100 × 200 × 500	120–240	–	–	–	US	Human spine	[79]
40 × 100 × 40	~23.5 *	7.6	–	128	US, PA	Human forearm	[80]

* Calculated from values provided in the literature. – not reported in the original publication.

In one of the initial studies, Huang et al. proposed a system that utilized a linear encoder along a 1-axis sliding track to record probe positions during manual scanning [69]. As the probe moved along the track, 2D B-mode images were acquired and stacked based on their spatial locations to generate 3D volumes. This method demonstrated feasibility by producing volumetric images of the human forearm. However, the fixed linear trajectory limited the scanning flexibility, reducing its suitability for imaging anatomies with curved or irregular surfaces.

To overcome this limitation, optical tracking systems have been widely adopted. Obst et al. developed a setup using five cameras and four reflective markers affixed to the US probe to track its position and orientation in real time [70]. The feasibility of the system was verified by scanning the Achilles tendon in humans. During manual scanning, the B-mode images and positional data were synchronized at 40 Hz, allowing for the 3D reconstruction of tendon morphology over a scanning range exceeding 120 mm (Figure 5b). Chung et al. further enhanced tracking accuracy using eight high-resolution digital cameras and smaller reflective markers attached to the LAT [71]. Their system achieved high spatial and temporal resolution, capturing B-mode images at a frame rate of 26 Hz with real-time positional tracking. Three-dimensional imaging of carotid artery phantoms showed a reconstruction error below 0.2 mm. Verhoef et al. extended this approach to achieve 3D UFD imaging of cerebral and tumor blood flow [72]. Volumetric data were acquired by scanning a LAT, while plane wave US images were acquired with 10 steering angles. A block-wise SVD and normalized convolution algorithms were applied to reconstruct 3D voxel-based images (Figure 5c). While the optical tracking method successfully demonstrated 3D image generation, accurate motion tracking requires line-of-sight visibility, which may limit usability in a clinical environment.

Electromagnetic tracking (EMT) systems offer an alternative that does not require line-of-sight. Cheung et al. employed EMT-based freehand scanning for spinal imaging [73]. During the freehand scanning, EMT recorded spatial data to map 2D frames into 3D space (Figure 5d). The reconstructed volume enabled curvature measurements of the human spine, showing a high correlation with X-ray-based Cobb angles (R^2^ = 0.86) (Figure 5e). However, slight underestimation of the curvature was observed, possibly due to tissue deformation. Lee et al. proposed an EMT-based 3D imaging system for quasi-static elastography that estimated tissue stiffness by computing displacements under mechanical compression [74]. In their method, a LAT probe was manually scanned across an elasticity phantom while applying repeated axial compression. EMT recorded the position and orientation of each B-mode frame during the scan, and the displacement and strain were calculated using a correlation-based algorithm. The registered 2D strain maps were reconstructed into a 3D elastogram, demonstrating accurate volumetric stiffness estimation for potential elastography applications.

To simplify the hardware setup, inertial measurement units (IMUs) have also been used. IMUs consist of integrated sensors such as accelerometers and gyroscopes to determine probe orientation. Herickhoff et al. employed an IMU to track orientation during the rotational and tilting motions of an imaging probe fixed to a custom-designed fixture [75]. Quaternion-based rotation data were assigned to each B-mode frame to reconstruct 3D orientation. However, this method lacked position tracking and could not compensate for probe or target displacement. Kim et al. addressed this limitation by integrating a distance sensor with the IMU to measure real-time axial displacement [76]. This allowed for the spatial registration of each image frame and significantly reduced misalignment artifacts. Their method enabled smoother surface rendering and improved anatomical continuity in reconstructed images of a breast phantom.

Beyond hardware-based tracking, software-based motion estimation methods have emerged to reduce system complexity. Prevost et al. proposed a machine-learning-based 3D reconstruction method [77]. A convolutional neural network (CNN) was trained to predict inter-frame displacement vectors between consecutive frames (Figure 5f). The model demonstrated robust performance across phantom and in vivo datasets, generating high-quality volumetric reconstructions under varied scanning conditions (Figure 5g). Similarly, Lyu et al. proposed a hybrid approach integrating optical tracking and software interpolation techniques for freehand scanning [78]. They applied Bezier interpolation and pixel nearest neighbor (PNN) methods to reconstruct images from spatially tracked data. Phantom and human experiments yielded mean spatial errors below 0.56 mm, accurately delineating key anatomical structures such as the thyroid and carotid artery. Additionally, Chen et al. enhanced EMT-based 3D ultrasound reconstruction through software techniques such as principal component analysis and discriminative scale space tracking [79]. They applied their method to spine imaging in adolescent idiopathic scoliosis patients, enabling the automatic detection and quantification of spinal deformities. The system achieved a high detection accuracy of 99.5%, highlighting the effectiveness of software-assisted motion correction in clinical applications. More recently, Lee et al. introduced a deep learning framework using global–local self-attention to improve freehand volumetric scanning [80]. They trained a deep learning model using US B-mode images. The resulting 3D volume showed effective motion tracking with strong generalizability. While the model is yet to be validated in the forearm, this technique can be applied to various regions of human applications. In another recent study, Luo et al. proposed an enhanced motion network that combines sensor-based tracking and image-based reconstruction for freehand 3D US volumes [81]. They integrated measurements from IMU and EM positioning systems with a temporal multibranch structure to refine motion estimation. The proposed approach was validated on human forearm, carotid, and thyroid datasets, showcasing its higher reconstruction accuracy compared to the existing methods.

## 5. Conclusions

LATs are among the most standardized and widely adopted probes in clinical ultrasound, routinely used to visualize anatomical structures in the breast, thyroid, tendons, blood vessels, and other superficial tissues. Although conventionally limited to 2D imaging, LATs can be extended to 3D imaging through various scanning mechanisms, each offering unique trade-offs in acquisition speed, flexibility, and clinical feasibility.

Among these techniques, motorized linear translation provides a straightforward method for volume reconstruction, enabling the accurate spatial stacking of B-mode images due to well-defined probe positioning. However, its rigid scanning path limits adaptability to curved or irregular anatomical surfaces. Robotic arm-based scanning addresses this limitation by providing programmable and repeatable probe control with high spatial precision, allowing scanning over complex surfaces. However, these advantages come with increased system complexity, higher cost, and reduced portability.

Freehand scanning, when combined with position-tracking systems such as optical, electromagnetic, or inertial sensors, provides the greatest operational flexibility for exploring anatomical regions without mechanical constraints. Optical tracking provides high spatial accuracy but requires a clear line-of-sight. Electromagnetic tracking avoids visibility issues but can be affected by metallic interference and environmental noise. IMU is compact and cost-effective, but is prone to drift motion, often requiring sensor fusion or periodic recalibration. All tracking methods can introduce errors such as misalignment, motion artifacts, or resolution loss in the reconstructed volume.

Recently, artificial intelligence (AI)-based reconstruction techniques have shown considerable promise in overcoming these issues by estimating motion directly from image data, correcting spatial errors, reducing noise, and improving volume continuity. These methods could reduce dependence on complex tracking hardware and enable robust volumetric imaging. To ensure reliability across different anatomies and scanning conditions, large and diverse training datasets are required.

Despite the potential of 3D USI, the clinical integration of 3D USI remains limited. One promising application is in automated breast volume scanning [82,83,84,85,86], where high-channel-count LATs (e.g., 768 elements) have been used in motorized scanning modules to acquire breast volumes for tumor detection, especially in dense breasts where mammography is less effective [87,88,89]. These systems are under active investigation for triaging breast nodules in clinical screening workflows. Similarly, 3D USI has shown utility in neuromuscular diagnostics through quantitative muscle ultrasound, with freehand scanning facilitating user-friendly acquisition for tracking disease progression in clinical practice [90,91,92,93].

A particularly promising direction involves integrating functional imaging capabilities with structural 3D USI. PAI, which offers molecular contrast based on optical absorption properties, has been successfully combined with LAT-based probes to enable dual-modal imaging systems. These integrated platforms have been validated in clinical studies across various applications, including thyroid nodules [94,95,96], breast tumors [97,98,99,100,101,102,103], and skin diseases [104,105,106]. Such systems provide not only volumetric anatomical information, but also molecular and hemodynamic biomarkers that may support improved disease characterization and personalized treatment planning. To date, most implementations of 3D PAI have relied on basic linear translational scanning mechanisms. However, by leveraging advanced scanning strategies, such as robotic-assisted, freehand-tracked, or multi-angle tomographic techniques, as reviewed in this paper, the scope and applicability of 3D PAI could be significantly broadened.

In addition to hardware innovations, advancements in reconstruction algorithms would enable the clinical adoption of 3D USI. Particularly in freehand scanning, accurate estimation of the spatial trajectory is essential for precise volume rendering. However, sensor drift, motion artifacts, and environmental interference often degrade image quality. AI-based methods would offer promising solutions for correcting tracking errors, enhancing image reconstruction, and enabling the real-time or automated interpretation of 3D data [107].

Furthermore, the automated tracking and restoration of continuous anatomical structures, such as blood vessels, will be critical for improving the completeness and diagnostic value of both US and PA volumes. Approaches such as vessel centerline extraction [108,109], volumetric segmentation [110,111], and model-driven reconstruction [112,113] can enforce spatial continuity across sequential frames or viewpoints and mitigate artifacts arising from limited-view geometry or acoustic shadowing.

Recent advances in high-frequency thin-film US sensors have expanded the capabilities of volumetric imaging by providing broader bandwidth and higher sensitivity [114]. These devices, often fabricated using micromachined materials, can operate at frequencies exceeding conventional US transducers, enabling higher spatial resolution and the improved detection of small anatomical features [115,116]. However, the reduced penetration depth inherent to high-frequency operation may constrain their widespread clinical application. Hybrid approaches that combine high-frequency thin-film transducers with conventional lower-frequency transducers could help balance resolution and depth, enabling both detailed superficial imaging and broader anatomical coverage [117,118].

Moving forward, several challenges remain for widespread clinical deployment. These include reducing system complexity and cost, improving user interface and ergonomics, minimizing acquisition time, and standardizing quality assurance protocols. Furthermore, robust clinical validation through large-scale studies will be essential to establish diagnostic accuracy, reproducibility, and clinical utility across diverse applications.

In conclusion, this review provided a comprehensive analysis of 3D USI systems based on LATs, highlighting key developments across scanning mechanisms, volume reconstruction strategies, and clinical applications. The reviewed technologies have demonstrated the feasibility of volumetric anatomical and functional data in both preclinical and clinical settings. With continued advancements in image acquisition, motion tracking, and intelligent reconstruction algorithms, 3D USI holds substantial promise as a next-generation tool for the quantitative and multi-dimensional assessment of biological tissues in a broad range of clinical applications.

## Data Availability

Not applicable.
